# Spasms from Intestinal Irritation and Teething

**Published:** 1862-10

**Authors:** Asahel Clark


Spasms from Intestinal Irritation and Teething.—*By Asahel Clark, M. D.*—In No. 11, I find a private letter from "G. C." that interested me very much, as I have had a case to treat, similar in some respects to the one described in that letter—a good description of which I have not yet found in any medical book. The little patient I speak of is a grandson of mine, and the case has perplexed me exceedingly. He is twenty months old; parents healthy, and this is their second child. He was weaned when about thirteen months old. Nothing occurred worthy of note till he was about seven months old, when he had a spasm while sitting in his little high-chair at the breakfast table. It soon passed off, and he appeared as well as usual during the rest of the day and night. The next morning at about the same hour, he had another while he was being dressed, and for several mornings in succession he had a spasm within twenty or thirty minutes of the same hour, but no more during the twenty-four hours. After about a week he had only one or two a week for three or four weeks, and then for ten or twelve days had several—one day having as many as twelve in the twenty-four hours,—some of them being very severe, and becoming more nearly epileptic in their character. Generally there was no frothing at the mouth, and he rallied in a very few minutes after the spasm subsided. About the eighth week, under the use of fluid extract of valerian and tonics, the spasms became less frequent and less severe, and about the ninth week had no spasms, and seemed to be doing well. He had no more spasms till he was about twelve months old—had one or two a week at first, but before the end of a month he had them every day, and some times five or six in a day. After three or four weeks they became less frequent, and he would pass three or four days without spasms.

It is now about four months since he has had a severe spasm, but has had "spells" such as "G. C." describes in his letter, every day, and some of the time as many as ten or twelve in a day, till within the last three weeks, during which time he has had none of those spells, and seems to be quite well. When these spells were coming on, he would look and reach for help, and on taking him he would cling close to the person taking him, draw up his feet and knees, utter four or five violent moans, (not screams exactly,) heart beating rapidly, and pupils dilated, the spell lasting but a few seconds, and passing off with a few full and rapid inspirations and expirations, and is then ready to engage in play again, or finish his meal. Violent, lancinating pain in abdomen I should suppose would produce just such motions, and just such moans as he makes when he has those spells.

As to treatment, I have tried a variety of remedies, and have had three of our best physicians in consultation. He has taken at three or four different times (when he was having spasms), calomel, followed by castor oil and spirits turpentine—followed by anodynes, laudanum, McMunn's elixir valerian, assifœtida (in the form of Dewees' Colic Mixture), etc. When he was about fifteen months old, and was having the spasms more violently, and becoming more epileptic in character, I gave him stramonium, and kept his pupils moderately dilated with it for ten days, without any effect upon the spasms; gave him Cannabis Indica two days, till it produced its specific effect, (unnatural laughing and merriment), but no modification of the spasms. He has inhaled chloroform whenever he has had spasms, from first to last. About six weeks ago he commenced taking arsenic, (Fowler's Solution 1, laudanum 1, and whisky 2, is the form I gave it, for the sake of convenience in dropping accurately.) Three drops of the above (three-fourths drop of the arsenical solution), and ten drops of the fluid extract of valerien, given together, three times a day, increasing the dose at the end of a week. While taking the above those spells became less frequent, and in about three weeks entirely disappeared. If "G. C.'s" little daughter has not recovered from those spells, ask him to try arsenic and valerian.

From the time my little grand-son was thirteen months old, and commenced having spasms the second time, he has hardly laughed, or even smiled often, but has cried and worried a great deal. Previous to that time he was unusually happy and playful—enjoyed a frolic and hearty laugh exceedingly. He is decidedly more mirthful and happy since he commenced taking the arsenic. He has *generally* been better at night than during the day time. Most of the time, even when he was having the spasms most severely, he has had tolerably good nights—seldom has he had a spasm in the night. During the day he has not slept as much and as soundly as most children of his age, but since he commenced taking the arsenic, he has more and better sleep in the day time. His stools have frequently been too light colored and curdy, and at such times he has taken small doses of calomel till the color of the stools was changed. Just before, or after the spasms or spells, there has generally been more or less gurgling in the bowels.

My opinion is that intestinal irritation and teething have been the cause of these spasms and spells in a great measure. Did not the "corn and apple-pudding," followed by severe exercise in the afternoon, have much to do in causing the spasms and spells in the case of "G. C.'s" little daughter? —*Buffalo Med. and Surg. Journal.*

